# Experimental Hydrocephalus Pre-meeting 7^th^ July 2010, Ike Barber Centre, University of British Columbia, Vancouver, Canada

**DOI:** 10.1186/1743-8454-7-S1-S2

**Published:** 2010-12-15

**Authors:** Hazel C Jones

**Affiliations:** 1 King’s College London, School of Biomedical and Health Sciences, London SE1 1UL, UK

## 

This year was a milestone since it was the 10^th^ anniversary of the first session on experimental studies on hydrocephalus held every year except one, since they were initiated in the SRHSB meeting in Atlanta, USA in year 2000. The aim has been to give researchers who are presenting experimental research at the main meeting, the opportunity to present their work in an informal atmosphere with time for feedback and useful discussion from the audience which usually amounts to 20 - 40 people.

The first speaker was Pat McAllister (University of Utah), who spoke on “*Behavioral and neuroimaging correlates of white matter injury in experimental hydrocephalus”*. He said that white matter tracts are known to be affected by ventriculomegaly and can be probed using diffusion tensor imaging (DTI), but not enough data have been accumulated to determine if this particular marker has reliable predictive power. He and colleagues have hypothesized that DTI and CSF pulsatility could serve as biomarkers to predict disease severity in experimental communicating hydrocephalus. This was investigated using their novel adult rat model of kaolin-induced communicating hydrocephalus, characterized by CSF flow impairments in the basal cisterns, marked changes in ventricular size, and variations in disease severity. Preliminary data has suggested that DTI changes in the corpus callosum and internal capsule *were* predictive of poor motor and cognitive outcomes, but that CSF pulsatility *was not* predictive. Ongoing analyses will correlate these non-invasive neuroimaging changes with specific cytopathology.

The second speaker was Weihong Yuan (Cincinnati Children’s Hospital) on “*Validation of diffusion tensor imaging as a biomarker for neonatal hydrocephalus”***.** He described an ongoing project designed to validate diffusion tensor imaging (DTI) as a biomarker to identify injury to the developing brain as a consequence of hydrocephalus. They propose to use DTI at 7 Tesla in an animal (rat) model of neonatal hydrocephalus and to compare results with outcome measures using histological examination of brain tissues and behavioral tests. The data obtained is expected to help to further understanding of pediatric hydrocephalus at the radiographic, pathologic and behavioral level.

The third talk was by Dorte Clemmensen and Mikkel Mylius Rasmussen (Aarhus University Hospital) on *“Tethered Cord – our first long term results in pigs”.* This year they presented their first long term survival results on six pigs that underwent either sham surgery (2) or kaolin-induced experimental tethered cord (4). At the point of sacrifice (3 months), magnetic resonance imaging showed what seemed to be an increased length and thinning of the medulla of the induced tethered cord animals when compared to sham. This study supports their view that the model is useful and development of the tethered cord is possible. However, the main goal, to achieve clinical symptoms of tethered cord syndrome, has yet to be accomplished.

After a coffee break, Andrew Baird (UCSD) spoke on behalf of a small network of five research groups that collaborate in hydrocephalus research between San Diego, Providence (Brown University) and the UK (University of Birmingham), on *“Can the Power of Zebrafish genetics be exploited to study hydrocephaly and CNS fluid balance?”* He discussed their joint efforts that include investigating whether a zebrafish model might help understand the role of factors produced by the choroid plexus and ependyma in hydrocephalus. After describing the many advantages and the significant disadvantages of a fish model, he emphasized that zebrafish have been extensively exploited by developmental biologists to understand the genetic basis for disease and that the results are now widely recognized as relevant to man, within limitations. Zebrafish can be used to selectively knockdown different genes *in vivo* including one they are studying called ECRG4 that produces augurin in the choroid plexus epithelium and ependyma. Knockdown ECRG4 produces a gene-dependant specific hydrocephalus-like phenotype that developmental biologists call CNS "edema". Because of its low cost, amenability to high throughput experimental analyses and its proven utility as a model for human diseases, the group propose that it should be considered as a complementary -- albeit not stand alone -- alternative to other animal models of hydrocephalus. If so, they believe it might be possible to ask very basic mechanistic, yet more physiological questions that cannot be addressed using either cells in culture or mammalian models of CNS injury and inflammation.

The next speaker was Alexander Shulyakov (University of Manitoba) who spoke on *“Brain biomechanics during acute obstructive hydrocephalus in live rats”.* He described how biomechanically, viscoelastic, nonlinear brain can accumulate strain with cyclic loading, in young more than in mature animals. In the normal state, intracranial pressure is maintained by vascular pulsation, and pulsating stress is mitigated by CSF oscillatory exits. In the case of CSF obstruction, due to Pascal’s principles, pulsating stress transmits undiminished to all parts of the enclosed fluid, resulting in a large multiplication of applied forces. CSF is a Newtonian (non compressible) fluid, the brain is viscoelastic, enabling accumulate strain (deformation) with cyclic, pulsating stress. Pulsating stress shrinks brain parenchyma (reducing extracellular space) leads to ventricular enlargement and hydrocephalus.

This talk was followed by Miles Miller (Brown University) who spoke on *“A study of human choroid plexus mRNA reveals disease-related changes in gene expression”.* To do this human lateral ventricle choroid plexuses from advanced Alzheimer's disease patients, healthy aged controls, and diseased controls (frontotemporal dementia and Huntington's disease) were obtained at autopsy. RNA was extracted and amplified, and human Affymetrix 48K gene arrays were utilized to investigate neurodegenerative disease-related changes in choroid plexus gene expression. The gene sets of greatest significance could be separated out into four experimental groups, revealing differences in choroid plexus gene expression when comparing the AD samples with both the normal and diseased control groups.

The final talk of the morning was by Mark Luciano and Steve Dombrowski (Cleveland Clinic) who spoke on *“Pulsatility and cerebral blood flow in hydrocephalus”.* They described how the movement of fluid through the cranial and spinal spaces appears to play a physiological role in cerebral compliance and therefore in blood flow. They suggest that the relationship between CSF flow and pressure pulsatility in altering cerebral blood flow is not clearly understood. In order to study the possible effect of cranial pulsatility on cerebral blood flow, they have developed a novel method and device to directly control CSF pulse amplitude via a cardiac-gated, oscillating bladder which dynamically modulates the CSF space, either accommodating or opposing entering systolic waves of blood. In an experimental model of hydrocephalus, it was possible to either augment or reduce intracranial pressure wave amplitude without affecting systemic conditions. Preliminary findings from this study suggest a relationship between CSF and CBF that may be involved in the underlying pathophysiology of hydrocephalus.

Overall, this was a very interesting session with lively presentations of novel research. Throughout the morning there were many discussions among presenters and audience participants that we hope were beneficial to all present.

